# Targeted screening of at-risk adults for acute HIV-1 infection in sub-Saharan Africa

**DOI:** 10.1097/QAD.0000000000000924

**Published:** 2015-12

**Authors:** Eduard J. Sanders, Elizabeth Wahome, Kimberly A. Powers, Lisa Werner, Greg Fegan, Ludo Lavreys, Clement Mapanje, R. Scott McClelland, Nigel Garrett, William C. Miller, Susan M. Graham

**Affiliations:** a Centre for Geographic Medicine Research – Coast, Kenya Medical Research Institute (KEMRI) – Kilifi, Kenya; b Nuffield Department of Medicine, University of Oxford, Headington, UK; c Department of Global Health, Academic Medical Centre, University of Amsterdam, Amsterdam, the Netherlands; d Department of Epidemiology, University of North Carolina at Chapel Hill, Chapel Hill, North Carolina, USA; e Centre for the AIDS Programme of Research in South Africa (CAPRISA), University of KwaZulu-Natal, Durban, South Africa; f Maisha Consulting bvba, Tildonk, Belgium; g UNC Project Malawi, Lilongwe, Malawi; h Departments of Medicine, Global Health, and Epidemiology, University of Washington, Seattle, Washington; i Department of Medicine, University of North Carolina at Chapel Hill, Chapel Hill, North Carolina, USA

## Abstract

**Background:**

Patients with acute HIV-1 infection (AHI) have elevated infectivity, but cannot be diagnosed using antibody-based testing. Approaches to screen patients for AHI are urgently needed to enable counselling and treatment to reduce onward transmission.

**Methods:**

We pooled data from four African studies of high-risk adults that evaluated symptoms and signs compatible with acute retroviral syndrome and tested for HIV-1 at each visit. AHI was defined as detectable plasma viral load or p24 antigen in an HIV-1-antibody-negative patient who subsequently seroconverted. Using generalized estimating equation, we identified symptoms, signs, and demographic factors predictive of AHI, adjusting for study site. We assigned a predictor score to each statistically significant predictor based on its beta coefficient, summing predictor scores to calculate a risk score for each participant. We evaluated the performance of this algorithm overall and at each site.

**Results:**

We compared 122 AHI visits with 45 961 visits by uninfected patients. Younger age (18–29 years), fever, fatigue, body pains, diarrhoea, sore throat, and genital ulcer disease were independent predictors of AHI. The overall area under the receiver operating characteristics curve (AUC) for the algorithm was 0.78, with site-specific AUCs ranging from 0.61 to 0.89. A risk score of at least 2 would indicate AHI testing for 5–50% of participants, substantially decreasing the number needing testing.

**Conclusion:**

Our targeted risk score algorithm based on seven characteristics reduced the number of patients needing AHI testing and had good performance overall. We recommend this risk score algorithm for use by HIV programs in sub-Saharan Africa with capacity to test high-risk patients for AHI.

## Introduction

During the first few weeks following HIV-1 acquisition, many people develop an acute retroviral syndrome (ARS), a set of nonspecific symptoms and signs, including fever, body pains, fatigue, or diarrhoea, for which urgent healthcare is frequently sought [1,2]. These symptoms on their own do not confirm acute HIV-1 infection (AHI) [3], but may help decide which patients need diagnostic evaluation for AHI [4–7]. Because patients with AHI would benefit from counselling and treatment to reduce onward transmission, approaches for targeted AHI screening are urgently needed.

AHI is the 2–4 week period before the development of detectable antibodies, when either p24 antigen or HIV-1 RNA testing is needed for diagnosis [2,8,9]. Infectiousness is sharply elevated during this period, making detection a priority for public health programming. Prompt diagnosis of AHI permits counselling for sexual risk reduction and the initiation of discussions about antiretroviral therapy (ART). ART has significant benefit for the reduction of transmission risk [10], and has recently been demonstrated to reduce morbidity and mortality in asymptomatic patients with CD4^+^ cell counts greater than 500 cells/µl [11,12].

Until recently, prospective screening of at-risk adults for AHI has been challenged by the absence of low-cost, rapid point-of-care (POC) p24 antigen or RNA assays in most clinical settings in resource-limited countries. In a year of low malaria transmission in coastal Kenya (2013), we diagnosed AHI through p24 antigen screening in 1.7% of febrile young adult patients seeking outpatient care and reported that AHI was as common as malaria in this population [13,14]. Given that POC diagnostics designed for AHI detection are emerging and may represent an opportunity for real-time AHI diagnosis in the near future [15], it is important to provide guidance on who should be targeted for AHI evaluation in resource-limited settings where universal screening is not feasible.

In high-prevalence countries such as Kenya and Malawi, two rapid tests are usually used in series or parallel to screen for HIV-1 antibodies. Among adults at high risk for HIV-1 acquisition who had discordant results on parallel rapid tests (i.e., one test positive, other test negative) the risk of subsequent HIV seroconversion was four- to 30-fold higher when compared with individuals from the same risk groups who tested HIV-1 negative on both antibody tests [5,7]. The World Health Organization recommends that patients with discordant rapid test results repeat HIV-1 testing 2 weeks later [16]. Ideally, such patients would undergo Nucleic Acid Amplification test testing at the time of serodiscordancy to expedite their diagnostic work-up and avoid delays in diagnosis.

Currently, no recommendations exist for patients who seek urgent care for fever or other ARS symptoms and have negative HIV antibody test results [14], despite three recent studies that have demonstrated the importance of AHI amongst febrile HIV-1-seronegative adult patients in Uganda, Kenya and Mozambique [14,17,18]. Published work on ARS has identified several symptoms and signs that are commonly reported across African sites [4–7]. However, published risk score algorithms have generally focused on a combination outcome including both AHI (defined as having negative HIV-1 antibody tests but positive p24 or HIV-1 RNA tests) and recent seroconversion, as patients with AHI were too few in any single study. Because guidelines already exist for the diagnosis and management of seroconverters [19], a risk score algorithm specifically developed to identify patients with AHI in diverse clinical settings could be valuable.

We therefore aimed to identify predictors of AHI using pooled data from four African studies and to assess the value of a risk score algorithm based on significant AHI predictors for use in targeting AHI screening.

## Methods

We pooled data from three cohort studies and one cross-sectional study that evaluated demographic factors, symptoms and signs compatible with ARS, and HIV-1 status among adult men and women at high risk for HIV-1 infection, in four different African settings. The three cohort studies involved female sex workers (FSW) in Mombasa, Kenya; MSM in Kilifi, Kenya; and women reporting sex work or multiple partners in the past 3 months in Durban, South Africa. The cross-sectional study recruited sexually transmitted diseases (STD) clinic patients in Lilongwe, Malawi. STD clinic patients in Malawi who were HIV-1 RNA positive but had negative or discordant rapid test results were followed for seroconversion. Detailed descriptions of the study settings and research methodologies have been previously described [4–7].

### Review of risk score algorithms to identify acute HIV-1 infection or recent seroconversion in Kenya, Malawi, and South Africa

Characteristics of the study populations, laboratory methods used, symptoms and signs ascertained, and predictors identified in these published studies are summarized in Table 1. Although no single predictor was identified at all four sites, fever and diarrhoea were associated with AHI or recent seroconversion in Mombasa, Kilifi and Lilongwe. Having discordant rapid HIV test results (evaluated only in Kilifi and Lilongwe) was the strongest predictor of the combined outcome across studies, as it was closely linked to seroconversion.

### Study procedures

In all four studies, procedures at study visits included a medical history to establish what symptoms had occurred since the previous visit (cohort studies), had preceded care seeking (cross-sectional study), or were present at the time of evaluation. In addition, a physical examination was conducted to confirm the presence or absence of selected signs, and a blood specimen was collected to evaluate patients for HIV-1 infection according to each study’s diagnostic algorithm (details in Table 1). Information on medical history and physical examination was collected blinded to HIV status.

### Selection of predictor variables for pooled analysis with acute HIV-1 infection outcome

For our pooled-data analysis focused on AHI as an outcome, we selected potential predictor variables that were collected by all four study sites. These included a total of 12 variables: age; sex; seven reported symptoms, including fever, diarrhoea, fatigue, headache, joint or muscle pain, sore throat, and rash; and three physical exam signs, including maculopapular rash, any lymphadenopathy, and genital ulcer disease (GUD). Each symptom and sign was coded in the dataset as present or absent.

### Definition of acute HIV-1 infection and documentation of seroconversion

AHI was defined by a positive HIV-1 RNA or p24 antigen test and two negative rapid antibody tests or ELISA assays, followed by documented antibody seroconversion. Patients with serodiscordant HIV-1 antibody tests at initial evaluation were also followed until seroconversion was documented. The estimated date of infection was determined as follows: less than 14 days before the collection date when the sample had a positive RNA viral load with negative p24 antigen and negative HIV-1 serology; 14 days before a positive p24 antigen test (with or without a positive RNA virus load) with negative HIV-1 serology; 18 days before discordant rapid HIV-1 antibody tests (with or without a positive p24 antigen test or positive RNA virus load); or the midpoint between a previously negative and subsequently positive HIV-1 serologic test (cohort studies), in the absence of a positive RNA viral load or p24 antigen test [20].

### Site-specific data

#### Mombasa

During 1993–1998, FSW were followed at monthly visits at the Ganjoni Municipal Clinic in Mombasa, Kenya, with 103 women acquiring HIV-1 [6]. For the pooled-data analysis, we used updated data from 1993 through 2014, which included data on 77 AHI cases. The following three predictors of AHI or recent seroconversion in Mombasa [6] were not collected at all four sites: too sick to work, inguinal lymphadenopathy, and candidiasis.

#### Kilifi

During 2005–2012, MSM were evaluated at monthly or quarterly visits, and 73 MSM acquired HIV-1 [7]. For the pooled analysis, we used updated data from 2005 through 2014, which included 20 AHI cases. The following predictor of AHI or recent seroconversion from Kilifi [7] was not collected at all four sites: any symptomatic STD (i.e., urethral or rectal discharge, or rectal pain).

#### Lilongwe

The dataset used to develop the published risk score algorithm was used [5], including data from 14 AHI cases. One predictor from the Lilongwe risk score algorithm (i.e., more than one sex partner in the past two months) was not collected at all four sites.

#### Durban

The dataset of monthly visits by high-risk women analysed for the published clinical algorithm was used [4], including data from 11 AHI cases. Three predictors from the Durban algorithm (i.e., loss of appetite, weight loss and vaginal discharge) were not collected at all four sites.

We created a variable called ‘‘body pains’’ to indicate the presence of joint or muscle pain, as a question about joint or muscle pain was asked at each of the four sites. In total, three symptoms and four signs identified as predictors of AHI or recent seroconversion at one or more sites were not collected by all four sites and therefore could not be included in the pooled-data analysis (indicated in Table 1).

### Choice of outcome

Although clinical guidance exists for patients who have HIV-1 serodiscordant or seropositive results [16], no guidance is available for how to identify and test HIV-1 antibody-negative patients in high-prevalence settings. In our pooled dataset, we included only AHI patient visits and control visits; data from seroconversion visits (at which one or both antibody tests was positive) were excluded from analysis. Data for AHI patients were censored at the AHI visit.

### Data analysis

To identify predictors of AHI in our pooled-data analysis, we calculated unadjusted prevalence odds ratios for medical history (i.e., symptoms) and physical examination findings (i.e., signs). We compared characteristics reported at AHI visits with those reported at all seronegative visits, using generalized estimating equation (GEE) with a logit link and exchangeable correlation matrix to adjust for intra-individual correlation. We constructed separate models for two specific domains (i.e., symptoms reported in past month and signs present on clinical examination), following the approach of Powers *et al.* [5]. Characteristics associated with AHI at *P* ≤ 0.15 in bivariate analysis were included in these separate, multivariable models for each of the two domains (i.e., symptoms and signs) [5]. We then constructed a combined model including age group, sex, site, and all variables with *P* ≤ 0.15 in each of the domain-specific models. The final pooled-data model retained only predictors associated with AHI at *P* ≤ 0.05 in this combined model.

### Predictor scores and score cut points

Similar to Powers *et al.* [5], each variable other than site in the pooled-data analysis received a predictor score equal to its beta coefficient (natural log of the adjusted prevalence odds ratio) from the final GEE model, rounded to the nearest integer. A risk score was then calculated for each study participant by summing the predictor scores for each predictor present at that visit. We then examined receiver operating characteristics curves to determine the optimal cut point of the calculated risk score, both overall and at each site. We also calculated the area under the curve (AUC) for the predictive ability of the pooled-data risk score algorithm to identify a patient visit at which AHI was diagnosed, overall and at each site.

### Comparison of acute HIV-1 infection visits and recent seroconverter visits

We were interested in whether there were significant differences in symptoms and signs at AHI visits (at which patients were antibody-negative but p24 antigen or HIV-1 RNA positive) and recent seroconverter visits (at which patients were positive on one or more HIV-1 antibody tests). We reasoned that such differences might be helpful in recognizing early symptoms of ARS. We therefore conducted a separate analysis comparing all AHI visits in our pooled-data analysis to the seroconverter visits we had excluded. Because patients evaluated for ARS several weeks after infection often have poor recall of symptoms [21], we restricted recent seroconverter visits to those conducted within 42 days of the estimated date of infection.

## Results

A total of 122 visits by AHI patients were compared with 45 961 uninfected patient-visits. There were differences in all symptoms and signs reported by AHI patients and by control participants across study sites (Table 2), except for reported rash and observed maculopapular rash. Kilifi MSM AHI patients had the highest report of any symptom (95%) and at least 60% reported fever, fatigue, headache, body aches or sore throat. Although 66% of Mombasa FSW AHI patients reported one or more symptoms, no single symptom was reported by over half of this group. Up to 50% of Lilongwe STD clinic AHI patients (12 men and 2 women) reported one or more symptoms, whereas only 27% of the high-risk women with AHI from Durban reported one or more symptoms.

The unadjusted prevalence odds ratios of age group, site, sex, and each of the seven symptoms and three signs are presented in the first column of Table 3. In the domain-specific model including all symptoms evaluated (column 3, upper box), fever, diarrhoea, fatigue, body pains, and sore throat were all associated with AHI at *P* ≤ 0.15. In the domain-specific model including all signs evaluated (column 3, lower box), only GUD was associated with AHI at *P* ≤ 0.15. Age 18–29 years, fever, diarrhoea, fatigue, body pains, sore throat, and GUD were all significantly associated with AHI (*P* ≤ 0.05) in the model combining demographic factors, symptoms, and signs (column 4), and so were retained in the final model. Predictor scores used for each variable in the final, pooled-data model are shown in Table 3, column 5. Of note, sex was not significant in the final model, and was thus not given points in the risk score algorithm. Site was included as an adjustment variable in the model, rather than as a predictor of AHI. Of note, site was not associated with AHI in the final adjusted model.

**Fig. 1:**
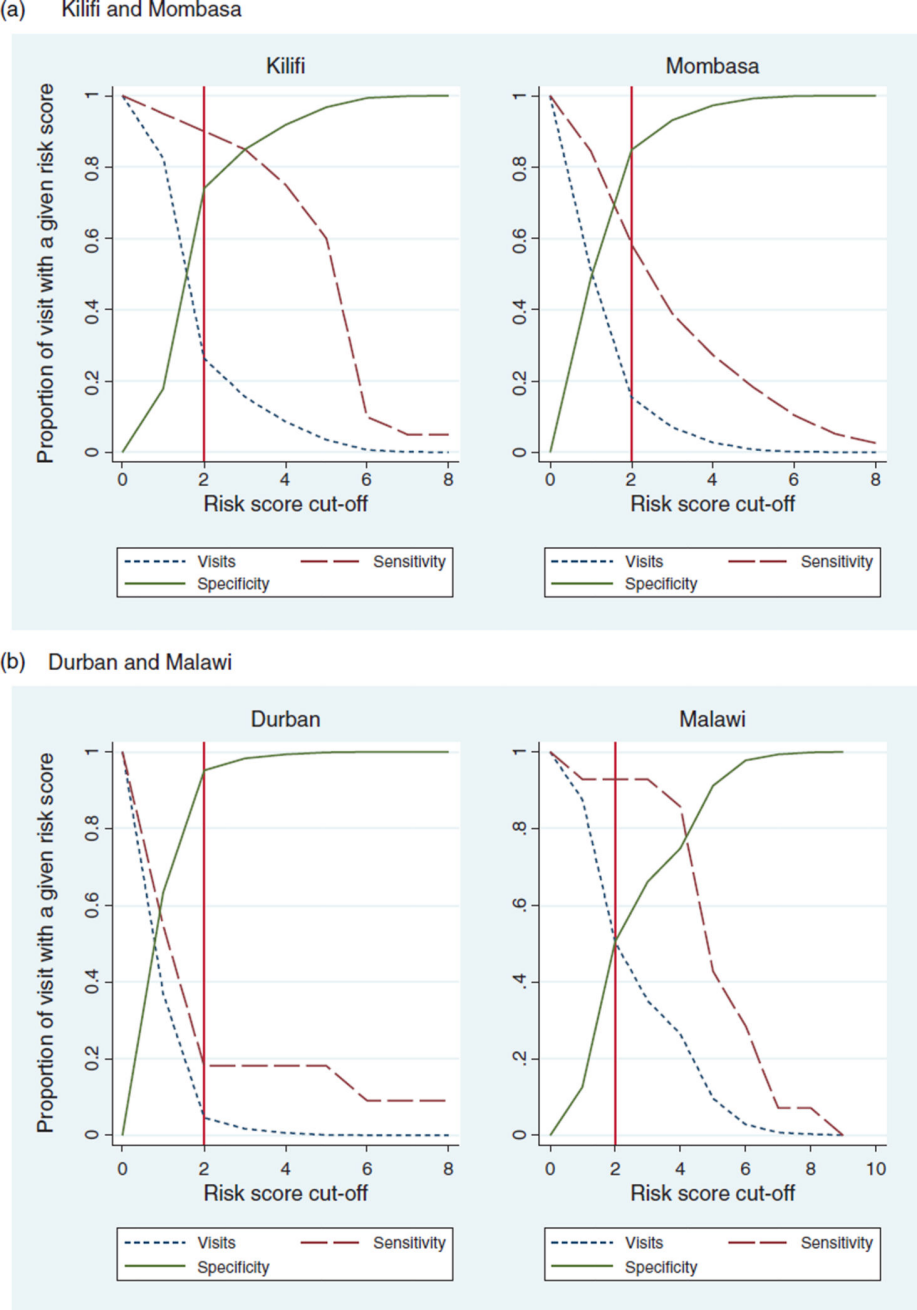
Proportion of visits at a given risk score at which AHI testing is indicated, and the corresponding sensitivity and specificity of the pooled-algorithm.

The overall AUC for the algorithm was 0.78, with site-specific AUCs of 0.77, 0.89, 0.83, and 0.61 for Mombasa, Kilifi, Lilongwe, and Durban, respectively. With a risk score cut-off of 2, two sites would have high sensitivity (90.0% in Kilifi, 92.9% in Lilongwe) and two would have moderate to high specificity (84.8% in Mombasa, 74.1% in Kilifi). In Durban, where over 70% of women were asymptomatic, a risk score cut-off of one would improve sensitivity. In Kilifi, and Malawi, a higher risk score cutoff of three and four, respectively, would improve specificity and decrease the proportion of patients needing AHI evaluation, at the expense of decreased sensitivity (Fig. 1). With a risk score cut-off of 2, the predictive abilities of the pooled-data algorithm when applied to each site’s data are shown in Table 4. A risk score of at least 2 using our pooled-data algorithm would indicate AHI testing for 15, 26, 50, and 5% of risk populations in Mombasa, Kilifi, Lilongwe, and Durban, respectively.

When we compared the 122 AHI visits with 148 visits by recent seroconverters at the four sites, the only significant difference was a higher report of GUD (21.3 vs. 4.7%, *P* < 0.02) at AHI visits compared with recent seroconverter visits (Supplemental Table 1, http://links.lww.com/QAD/A804). In Mombasa, comparing 77 AHI visits to 85 recent seroconverter visits, there was a higher report of fatigue (35.1 vs. 16.5%, *P* < 0.007) and GUD (17.3 vs. 2.4% respectively, *P* < 0.01). In Kilifi, comparing 20 AHI visits to 39 recent seroconverter visits, there was a higher report of sore throat (60.0 vs. 28.2% respectively, *P* < 0.02). For Lilongwe and Durban, where the numbers of AHI and recent seroconverter visits were limited, there were no statistically significant differences.

## Discussion

We assessed whether signs and symptoms identified in a pooled-data analysis predicted AHI in HIV-1 antibody-negative adults at high risk for HIV-1 acquisition in eastern and southern Africa. We pooled data from four study sites in Kenya, Malawi, and South Africa that ascertained symptoms, signs, and HIV-1 status over extended periods (>20 years in Mombasa, Kenya; and >9 years in Kilifi, Kenya). This analysis allowed us to identify demographic factors, symptoms, and signs associated with AHI, including age 18–29 years, fever, fatigue, body pains, diarrhoea, sore throat, and GUD. A risk score algorithm based on predictor coefficients had good performance in Kilifi and Lilongwe, fair performance in Mombasa, and weak performance in Durban. A new algorithm targeting AHI screening using p24 antigen or HIV-1 RNA tests to HIV-1-antibody-negative persons with a risk score of at least 2 could assist providers in African settings in identifying individuals with AHI, who are at very high risk for transmitting HIV-1 infection to their sexual partners.

What are the practicalities of this algorithm? It requires clinicians to complete a 7-item score card, capturing age and documenting the presence of any of the five reported symptoms or GUD on physical exam. Although all patients seeking care should be offered standard HIV counselling and testing per current guidelines, patients who test HIV-1 antibody negative and have a calculated risk score of at least 2 would undergo laboratory testing to detect AHI. This approach would allow efficient detection of AHI via RNA or p24 testing only in the subset of patients most likely to have AHI. Previously, we showed that 1% of adult patients aged 18–29 years who were targeted for AHI evaluation using a clinical algorithm and agreed to be tested had AHI in Coastal Kenya, whereas approximately 25% of patients seeking care were eligible for AHI evaluation [14]. Prospective studies to assess the yield of AHI patients diagnosed using our pooled-data algorithm to target AHI testing would provide useful information about the performance of the algorithm in additional settings.

Reports of symptoms and signs detected at clinical examination differed by study site in this analysis. In Durban, where there is a subtype C epidemic, the majority (73%) of higher risk women who acquired HIV-1 remained asymptomatic. Interestingly, most women and men who acquired HIV-1 in a large study of seroconversion among subtype C-infected discordant couples also remained asymptomatic [22]. Why such a high proportion of women in Durban reported no symptoms is unclear. Previously, we showed that patients infected with HIV-1 subtypes A, C, or D who participated in a multicentre AHI study in five African countries had differences in their ARS symptoms (i.e. subtype C or D had fewer ARS symptoms reported than subtype A) [23]. Although it remains to be elucidated if viral properties of transmitted HIV-1 subtypes or differences in innate immune response by subtype drive the range of symptoms seen in AHI [24], our risk score algorithm performed at least moderately well in three of four settings. A simple approach to AHI screening that targets symptoms and signs, when present, may be the most realistic strategy for AHI detection in all regions of sub-Saharan Africa, given cost constraints.

In order to reduce ongoing HIV-1 transmission, new strategies will be necessary to find at-risk adults with undiagnosed HIV-1 infection, including patients with AHI, and link them to care. Patients with AHI are the most difficult to diagnose, but can be identified using p24 antigen testing or HIV-1 RNA-based tests such as NAAT [14]. As new POC tests become available, research is urgently needed on which AHI detection strategies are most cost-effective, which may vary by site or programme. Patients diagnosed with AHI should undergo risk reduction counselling and counselling about the benefits of ART initiation, both for preventing transmission to their sexual partners and children, and in order to preserve immune function and prevent HIV-1-related morbidity and mortality. The cost-effectiveness of AHI detection should be compared with ongoing efforts to identify patients with prevalent HIV-1 based on antibody tests done at care seeking (i.e., provider-initiated or diagnostic testing and counselling). Unfortunately, providers currently miss testing opportunities, and may identify only those patients with the most advanced immunosuppression, who are most obviously in need of testing [19]. Outcomes of a combined strategy to test for prevalent and AHI should be modelled in terms of patients linked to care and secondary transmission prevented. The cost for AHI screening and the yield of AHI cases found will likely be determined by locally prevailing illnesses that present with similar symptoms (e.g., malaria, arboviral infections), by clinical setting (e.g., HIV-uninfected STD clinic patients in Lilongwe had the highest number of symptoms and signs), by gender (i.e., women appear to report fewer symptoms than men) [22,25], and by HIV-1 incidence in the target population.

Although our study is unique in the large number of AHI patients included in the pooled dataset from four study sites in Africa, it has several limitations. First, because we could only include variables collected at all four study sites in the pooled-data model, we may have missed one or more important predictors of AHI. Nevertheless, this would not have affected performance of the current risk score, which was moderate to very good overall and at three of the four sites. Second, some study sites had a particular clinical focus (i.e., on seroconversion illness or STD screening), and so may have been more thorough in their assessments of certain symptoms or signs. Finally, the at-risk populations included may not represent all patients who acquire HIV-1 in African settings; nevertheless, key populations are critical in any consideration of how to target testing for AHI to those at highest risk for HIV-1 acquisition.

Despite these limitations, our study shows that targeted screening of at-risk adults for AHI using a simple algorithm based on seven features (age 18–29 years, fever, fatigue, diarrhoea, body pains, sore throat, and GUD) would substantially reduce the number of HIV-1-seronegative patients requiring testing. Validation of the utility of this model at other African sites and wider access to POC HIV-1 RNA testing that enables sites to screen for AHI would help ensure that HIV-1 is diagnosed as early as possible, patients are promptly counselled and initiate ART, and that opportunities to reduce HIV-1 transmission are not missed.

## Supplementary Material

Supplemental Table 1

## Figures and Tables

**Table 1:** Predictors of acute HIV infection and recent seroconversion in Kenya, Malawi, and South Africa.

Site, country (first author, year publication)	Mombasa, Kenya (Lavreys, 2000)	Kilifi, Kenya (Wahome, 2013)	Lilongwe, Malawi (Powers, 2007)	Durban, South Africa (Mlisana, 2013)
Study design	Cohort	Cohort	Cross-sectional	Cohort
Risk group	female sex workers	MSM	Sexually transmitted diseases clinic patients	High-risk women
Number of AHI/seroconversion cases^a^	103	73	21	28
Number of visits	7735 visits	6531 visits	860 visits	4845 visits
Follow-up	Monthly	Monthly or quarterly	None	Monthly
Laboratory methods used for AHI and antibody detection	ELISA + WB	Dual rapid Ab and p24ag RNA confirmation	Dual rapid Ab, WB and RNA	Dual Ab and RNA
Significant predictors of AHI or recent seroconversion				
Socio-demographic and behavioural factors				
Age (18–24 years)				X
Age (18–29 years)		X		
>1 sex partner^b^ in the past 2 months			X	
Reported symptoms				
Fever	X	X	X	
Vomiting^b^	X			
Diarrhoea	X	X	X	
Fatigue		X		
Headache	X			
Body aches (arthralgia, myalgia)	X		X	
Sore throat				X
Weight loss^b^				X
Rash	X			X
Loss of appetite				X
Swollen lymph nodes	X			
Too sick to work^b^	X			
Any symptomatic STD^b^		X		
Physical exam signs				
Extra-inguinal lymphadenopathy at ≥2 sites^b^	X			
Inguinal lymphadenopathy at ≥2 sites^b^	X			
Genital ulcer disease (GUD)			X	X
Vaginal discharge^b^				X
Vaginal candidiasis^b^	X			
Discordant rapid test results		X	X	

AHI, acute HIV infection; STD, sexually transmitted disease; WB, Western Blot.

a All AHI patients and patients with only one positive antibody test were followed until documented seroconversion (i.e., two positive antibody tests).

b Predictor could not be included in the pooled-data analysis, as it was not collected at all four sites.

**Table 2:** Characteristics of acute HIV infection (AHI) and seronegative patient visits in Mombasa, Kenya; Kilifi, Kenya; Lilongwe, Malawi; and Durban, South Africa.

Characteristics Risk group	Mombasa, Kenya^a^, FSW		Kilifi, Kenya^a^, MSM		Lilongwe, Malawi, STD clinic patients		Durban, South Africa High-risk women		Comparison across study sites	Pooled estimate	
Number of AHI visits	77		20		14		11		*P*-value	122	
Socio-demographic characteristics	*n*	%	*n*	%	*n*	%	*n*	%		*n*	%
Age (18–29 years)	44	57.1	15	75.0	13	92.9	4	36.4	0.01	76	62.3
Symptoms											
Fever	36	46.8	15	75.0	7	50.0	3	18.2	0.02	60	49.2
Diarrhoea	7	9.1	7	35.0	2	14.3	2	18.2	0.04	18	14.8
Fatigue	27	35.1	16	80.0	2	14.3	2	18.2	<0.001	47	38.5
Headache	32	41.6	15	75.0	5	35.7	1	9.1	0.003	53	43.4
Body aches (arthralgia, myalgia)	26	33.8	17	85.0	6	42.9	2	18.2	<0.001	51	41.8
Sore throat	10	13.0	12	60.0	0	0.0	2	18.2	<0.001	24	19.7
Rash	3	3.9	2	10.0	1	7.1	2	18.2	0.3	8	6.6
Any symptom	51	66.2	19	95.0	7	50.0	3	27.3	0.001	80	65.6
Signs											
Maculopapular skin rash	0	0.0	1	5.0	0	0.0	0	0.0	0.2	1	0.8
Genital ulcer	13	16.9	1	5.0	11	78.6	1	9.1	<0.001	26	21.3
Any palpable lymph nodes	2	2.6	4	20.0	6	42.9	0	0.0	<0.001	12	9.8
Any sign	14	18.2	5	25	13	92.9	1	9.1	<0.001	33	27.1
Number of HIV-1-seronegative visits	33 321		7034		839		4844			45 961	
Socio-demographic characteristics											
Age (18–29 years)	33244	36.4	5051	71.8	634	75.6	1463	30.2	<0.001	19238	41.9
Symptoms											
Fever	5207	15.5	1063	15.1	184	21.9	140	2.9	0.02	6523	14.2
Diarrhoea	1023	3.1	429	6.1	34	4.1	102	2.1	0.04	1578	3.4
Fatigue	2273	6.8	975	13.8	74	8.8	133	2.8	<0.001	3431	7.5
Headache	5625	16.8	1317	18.7	216	25.7	277	5.7	0.003	7365	16.0
Body aches (arthralgia, myalgia)	2707	8.1	1305	18.5	144	17.2	166	3.4	<0.001	4283	9.3
Sore throat	1374	4.1	584	8.3	11	1.3	100	2.1	<0.001	2053	4.5
Rash	426	1.3	210	3.0	75	8.9	108	2.2	0.3	817	1.8
Any symptom	9805	29.3	2578	36.6	436	52.0	605	12.5	0.001	13306	29.0
Signs											
Maculo-papular skin rash	178	0.5	100	1.4	19	2.3	97	2.0	0.2	391	0.9
Genital ulcer	331	1.0	41	0.6	220	26.2	3	0.1	<0.001	590	1.3
Any palpable lymph nodes	1450	4.3	738	10.5	158	18.8	260	5.4	<0.001	2590	5.6
Any sign	1878	5.6	834	11.8	313	37.3	346	7.1	<0.001	3351	7.3

FSW, female sex worker; STD, sexually transmitted disease.

a For the two Kenya sites (Mombasa and Kilifi), datasets were updated to include additional visits beyond what was included in the published algorithm articles.

**Table 3:** Modelling of characteristics associated with acute HIV infection (AHI) in the pooled dataset.

Predictor	Unadjusted POR (95% CI)	Domain-specific model adjusted POR (95% CI)^a^	Combined model adjusted POR (95% CI)^b^	Final model adjusted POR (95% CI)^c^	Beta coefficient^d^	Score^e^
Socio-demographic						
Age (18–29 years)	2.2 (1.5–3.2)		2.1 (1.4–3.0)	2.1 (1.4–3.0)	0.7	1
Site						
Kilifi, Kenya	Ref		Ref	Ref		
Mombasa, Kenya	0.8 (0.5–1.4)		1.7 (0.3–8.9)	1.7 (0.3–8.9)		
Lilongwe, Malawi	5.8 (2.9–11.6)		2.0 (0.8–4.7)	2.0 (0.8–4.7)		
Durban, South Africa	0.8 (0.4–1.7)		2.9 (0.5–16.5)	2.9 (0.5–16.5)		
Sex	1.7 (1.1–2.6)		1.1 (0.2–5.4)	1.1 (0.2–5.4)		
Symptoms at evaluation visit						
						
Fever	5.7 (4.0–8.1)	1.9 (1.1–3.2)	2.2(1.4–3.5)	2.2(1.4–3.5)	0.8	1
Diarrhoea	4.7 (2.9–7.8)	1.4 (0.8–2.4)	1.8 (1.1–2.9)^e^	1.8 (1.1–2.9)	0.6	1
Fatigue	7.5 (5.2–10.8)	2.7 (1.6–4.8)	2.6 (1.6–4.2)	2.6 (1.6–4.2)	0.9	1
Head ache	3.9 (2.8–5.6)	1.2 (0.7–2.1)	–	–		
Body pains	6.8 (4.7–9.7)	1.8 (1.1–2.9)	2.3 (1.5–3.4)	2.3 (1.5–3.4)	0.8	1
Rash	3.7 (1.8–7.6)	1.5 (0.6–3.3)	–	–		
Sore throat	5.0 (3.2–7.9)	1.5 (0.9–2.6)	1.7 (1.0–2.8)	1.7 (1.0–2.8)	0.5	1
						
Signs at evaluation visit						
						
Rash	3.7 (1.8–7.6)	0.7 (0.6–3.3)	–	–		
Any palpable lymph nodes	6.8 (3.7–12.7)	1.1 (0.6–2.1)	–	–		
Genital ulcer	20.0 (12.9–31.1)	19.8 (12.5–31.3)	14.9 (8.6–26.0)	14.9 (8.6–26.0)	2.7	3
						

CI, confidence intervals; POR, prevalence odds ratio.

a Factors associated with AHI at *P* ≤ 0.15 were included in two separate multivariable models for two domains: ‘symptoms’ and ‘signs’ findings. Each model is presented in this column, with a box indicating the results for each model.

b Factors associated with AHI at *P* ≤ 0.15 in the initial domain-specific models were included in a combined model.

c All variables in the final model, with the exception of sex or site, were associated with acute HIV infection at *P* ≤ 0.05.

d Natural log of the adjusted prevalence odds ratio of the final model.

e Predictor score is equal to its beta coefficient (natural log of the adjusted prevalence odds ratio) from the final generalized estimating equation model, rounded to the nearest integer.

**Table 4:** Performance of the final pooled-data algorithm with a risk score of at least 2 when applied to site-specific data from Mombasa, Kilifi, Lilongwe and Durban.

Predictive ability	Mombasa, Kenya FSW	Kilifi, Kenya MSM	Lilongwe, Malawi STD clinic patients	Durban, South Africa high-risk women
Sensitivity	58.4%	90.0%	92.9%	18.2%
Specificity	84.8%	74.1%	50.5%	95.4%
Positive predictive value	0.9%	1.0%	3.0%	0.9%
Negative predictive value	99.9%	100.0%	99.8%	99.8%
AUC	0.77	0.89	0.83	0.61
AHI testing indicated	15.3%	26.1%	50.2%	4.6%

AHI, acute HIV infection; AUC, area under the curve; STD, sexually transmitted diseases.
