# Supplementary material for: Targeted screening of at-risk adults for acute HIV-1 infection in sub-Saharan Africa
Source: AIDS. Author manuscript; Available in PMC 2016 Jan 15. (PMC4714928; doi:10.1097/QAD.0000000000000924)
Supplement: Supplemental Table 1 [file NIHMS748732-supplement-Supplemental_Table_1.docx]

| Characteristics of study population | Mombasa, Kenya  FSW  N=162 | | | | Kilifi, Kenya  MSM  N=59 | | | | Lilongwe, Malawi  STD clinic patients  N=21 | | | | Durban, South Africa  High-risk women  N=28 | | | | Total  N=270 | | | | |
| --- | --- | --- | --- | --- | --- | --- | --- | --- | --- | --- | --- | --- | --- | --- | --- | --- | --- | --- | --- | --- | --- |
|  | **AHI^1^** | | **EHI^2^** | | **AHI** | | **EHI** | | **AHI** | | **EHI** | | **AHI** | | **EHI** | | **AHI** | | | **EHI** | |
| N | 77 | | 85 | | 20 | | 39 | | 14 | | 7 | | 11 | | 17 | | 122 | | | 148 | |
| Time EDI^3^ - ascertainment (days) | <15 | | 19-42 | | <15 | | 19-42 | | n/a | | n/a | | <15 | | 19-42 | | <15 | | | 18-42 | |
| Symptoms |  |  |  |  |  |  |  |  |  |  |  |  |  |  |  |  |  |  | |  |  |
| Fever | 36 | 46.8 | 44 | 51.8 | 15 | 75.0 | 24 | 61.5 | 7 | 50.0 | 5 | 71.4 | 3 | 18.2 | 2 | 17.7 | 60 | 49.2 | | 76 | 51.4 |
| Diarrhoea | 7 | 9.1 | 7 | 8.2 | 7 | 35.0 | 10 | 25.6 | 2 | 14.3 | 2 | 28.6 | 2 | 18.2 | 1 | 5.9 | 18 | 14.8 | | 20 | 13.5 |
| Fatigue | 27 | 35.1* | 14 | 16.5* | 16 | 80.0 | 23 | 59.0 | 2 | 14.3 | 2 | 28.6 | 2 | 18.2 | 5 | 29.4 | 47 | 38.5 | | 44 | 29.7 |
| Headache | 32 | 41.6 | 38 | 44.7 | 15 | 75.0 | 21 | 53.9 | 5 | 35.7 | 3 | 42.9 | 1 | 9.1 | 5 | 29.4 | 53 | 43.4 | | 67 | 45.3 |
| Body aches | 26 | 33.8 | 22 | 25.9 | 17 | 85.0 | 24 | 61.5 | 6 | 42.9 | 4 | 57.1 | 2 | 18.2 | 4 | 23.5 | 51 | 41.8 | | 54 | 36.5 |
| Sore throat | 10 | 13.0 | 11 | 12.9 | 12 | 60.0* | 11 | 28.2* | 0 | 0.0 | 1 | 14.3 | 2 | 18.2 | 4 | 23.5 | 24 | 19.7 | | 27 | 18.2 |
| Rash | 3 | 3.9 | 7 | 8.2 | 2 | 10.0 | 2 | 5.1 | 1 | 7.1 | 1 | 14.3 | 2 | 18.2 | 3 | 17.7 | 8 | 6.6 | | 13 | 8.8 |
| Swollen lymph nodes | 4 | 5.2 | 3 | 3.5 | 7 | 35.0 | 8 | 20.5 | - |  | - |  | 0 | 0 | 2 | 11.8 | 11 | 10.2 | | 13 | 9.2 |
| Signs |  |  |  |  |  |  |  |  |  |  |  |  |  |  |  |  |  |  | |  |  |
| Maculopapular skin rash | 0 | 0.0 | 0 | 0.0 | 1 | 5.3 | 3 | 7.7 | 0 | 0.0 | 0 | 0.0 | 0 | 0.0 | 2 | 11.8 | 1 | 0.8 | | 5 | 3.4 |
| Genital ulcer | 13 | 17.3* | 2 | 2.4* | 1 | 5.3 | 0 | 0.0 | 11 | 78.6 | 4 | 57.1 | 1 | 9.1 | 1 | 5.9 | 26 | 21.3* | | 7 | 4.7* |
| Any palpable lymph nodes | 2 | 9.5 | 11 | 32.4 | 4 | 20.0 | 9 | 23.1 | 6 | 42.9 | 5 | 71.4 | 0 | 0.0 | 1 | 5.9 | 12 | 9.8 | | 26 | 17.6 |
| Discordant rapid test results | 0 | 0.0 | 6 | 7.1 | 0 | 0.0 | 8 | 20.5 | 0 | 0.0 | 7 | 100.0 | 0 | 0.0 | 1 | 5.9 | 0 | 0.0 | 22 | | 14.9 |

1). Acute HIV-1 infection (AHI) was defined by a positive PCR or p24 antigen test and negative rapid antibody tests or ELISA, in patients who subsequently had a documented seroconversion.

2). Early HIV-1 infection (EHI) was defined by documented antibody seroconversion, or discordant rapid test or ELISA results with subsequent seroconversion (Kenya and South Africa); or discordant rapid tests and negative or indeterminate Western blot; or discordant rapid tests and weakly positive Western blot with subsequent band evolution (Malawi).

3). Estimated date of infection for comparison of AHI vs. EHI groups within the site.

*P<0.05
